# Study on Permeability Coefficient of Saturated Clay Modified by Fractal Theory and Poiseuille Theory

**DOI:** 10.3390/ma19010021

**Published:** 2025-12-20

**Authors:** Lu Guo, Xiaoyang Xin, Keqiang He

**Affiliations:** 1 School of Civil Engineering and Architecture, Suqian University, Suqian 223800, China; 2 Pingdingshan Zhao Pingtai Reservoir Operation and Support Center, Pingdingshan 467000, China; 3 School of Civil Engineering, Qingdao University of Technology, Qingdao 266520, China; keqianghe@qut.edu.cn

**Keywords:** saturated clay, permeability coefficient, Poiseuille law, effective specific surface area, effective void ratio

## Abstract

The permeability coefficient of saturated clay plays a crucial role in practical engineering applications. In this paper, based on the fractal geometry theory and combined with the relationship between the flowing water volume and non-flowing water volume in saturated clay, the theoretical formulas for the effective pore specific surface area and the effective void ratio of saturated clay are established. Based on the capillary seepage channel model of saturated clay, combined with Poiseuille’s law and the concept of equivalent hydraulic radius, the theoretical formula for the permeability coefficient of saturated clay is established. Finally, the physical parameters of the remolded clay samples are measured and substituted into the modified Kozeny–Carman equation and the equivalent capillary seepage equation of saturated clay before and after the modification. Through the comparative analysis of the above theoretical values and the measured values of indoor seepage tests, it is found that the saturated clay seepage equation established in this paper is more suitable for dense saturated clay with relatively small pores. It has the characteristics of higher calculation accuracy and easier acquisition of basic parameters. The research results provide important references for practical engineering and the study of saturated clay seepage theory, and have broad prospects for practical engineering applications.

## 1. Introduction

With the rapid development of economic construction, the engineering geological problems related to the pore water permeability of clayey soil caused by human engineering activities are becoming increasingly serious. The law of pore water permeability of clayey soil has become the fundamental and key issue for solving these problems. In early engineering applications, saturated clay was often regarded as an impermeable layer because its permeability is much lower than that of sandy soil. However, with the frequent occurrence of a series of engineering problems, such as ground subsidence and deformation, slope sliding and instability with soft-clay interlayers, seepage of earth–rock dams with clay core walls, and sudden gushing in foundation-pit tunnels, etc., the study of seepage in clay has attracted widespread attention in the engineering field and academic circles [1,2,3,4]. For cohesionless soil such as sand and gravel, the number and scale of its internal pores are relatively large, and the electric field force on the surface of soil particles has little influence on the seepage water, so the seepage of cohesionless soil is highly consistent with the macroscopic seepage principle of water. The Darcy percolation law and Dupuit percolation principle have been highly recognized by engineers and scholars after many years of practical engineering tests [5,6]. However, due to the small size of clay particles and the main mineral compositions being montmorillonite, illite, and kaolinite, isomorphous replacement occurs in the deposition process, which makes the surface of clay particles produce negative charge and absorb cations and polar water molecules in water. Then, the bound water film is formed. The tiny clay particles are stacked to form a sheet or sheet contact structure under the action of Coulomb forces, Van Der Waals force, and gravity. When clay particles form a sheet-like interface, the pores are small, and the pores are completely filled with combined water. Therefore, invalid unconnected pores such as “blind hole”, “end hole”, and “solitary hole” are formed, which leads to the complexity and diversity of the water seepage mechanism in clay. Because the permeability coefficient is closely related to many factors, such as porosity, saturation, hydraulic gradient, fluid type, and soil structure, it is more difficult to accurately determine the permeability coefficient of saturated clay. In recent years, many scholars at home and abroad have focused on the factors such as clay porosity, saturated water content, effective specific surface area, initial hydraulic gradient, fluid type, and soil structure, and established many modified seepage theoretical models on the basis of the Darcy law and the Kozeny–Carman equation. Mesri and Olsonr (1971), Raymond (2011), and Horpibulsuk et al. (2011) have found the initial head phenomenon in clay seepage, which deviates from the linear relationship between velocity and hydraulic gradient in Darcy’s law [7,8,9]. In clay seepage, the permeability coefficient *k* and the parameter *e*^3^/(1 + *e*) (where *e* denotes void ratio) do not exhibit a linear relationship. Consequently, new equations have been proposed to characterize seepage behavior in fine-grained soils. However, these formulations demonstrate superior performance only when applied to specific soil types. Reddi and Thangavadivelu (1996) idealized the clay structure into a spherical cluster structure, and constructed an idealized permeability theoretical model of a clay spherical cluster structure, but the pore size and structure of clay are not considered in the model [10]. When the hydraulic gradient is small, the resulting error is large. Chapuis and Aubertin (2003), Maček et al. (2013), and Kobayashi et al. (2017) analyzed the characteristics of the testing methods for the specific surface area of different soils, and discussed the value of the specific surface area in the Kozeny–Carman equation [11,12,13]. Aimrun et al. (2004), Sun et al. (2016), Fang et al. (2020), and Liu et al. (2021) found that the adsorbed bound water on the clay surface could not flow and occupied the volume of the effective seepage pores [14,15,16,17]. And combined with clay structure and statistical theory, the comprehensive constant of Kozeny–Carman seepage model and the specific surface area and void ratio of clay particles are modified. But it generally has the defect that it is difficult to determine the parameters, so it cannot be widely used in the practical engineering field. Singh and Wallender (2008), Liang and Fang (2010), and Zhang et al. (2024) have studied the microstructure and physical and mechanical properties of saturated clay from a microscopic perspective, and established a theoretical estimation model of equivalent permeability coefficient of saturated clay based on the microscopic law of saturated clay; however, the characteristics of clay are greatly affected by factors such as region and stress history, resulting in great differences in the characteristics of micropores, so the above model has the deficiency of a narrow applicable area [18,19,20].

The above research shows that although many correction models have been proposed by experts and scholars at home and abroad, there is no accurate theoretical calculation formula for the Kozeny–Carman constant yet. It is only a comprehensive empirical value without practical physical significance, and the seepage mechanism of saturated clay cannot be accurately described. Therefore, the calculation accuracy of the modified Kozeny–Carman equation is low. Based on the particle size and pore size characteristics of clay, this paper uses the basic physical and mechanical parameters of saturated clay, conducts deductions based on seepage theory, and combines the equivalent seepage capillary bundle theory of saturated clay, Poiseuille’s law, and fractal theory to establish a theoretical calculation formula for the clay permeability coefficient. This formula is convenient for engineering applications and has definite physical and mechanical significance.

## 2. Theoretical Calculation Formula of Permeability Coefficient of Saturated Clay

### 2.1. Theoretical Calculation Formula of Permeability Coefficient of Saturated Clay Based on Correction of Effective Void Ratio and Effective Specific Surface Area

**(1)**  **Hypothetical condition**

The pores in clay are tortuous and complex. There are not only connected pores but also a large number of ineffective unconnected pores. In saturated clay, there is plenty of bound water and free water, and only free water and part of the weak bound water are involved in seepage. The resistance of seepage flow in saturated clay is mainly provided by the dynamic viscous resistance of water and the additional viscosity produced by the Van Der Waals force between the surface of soil particles and water molecules. Moreover, the seepage flow in saturated clay is continuous and interactive.

**Figure 1:**
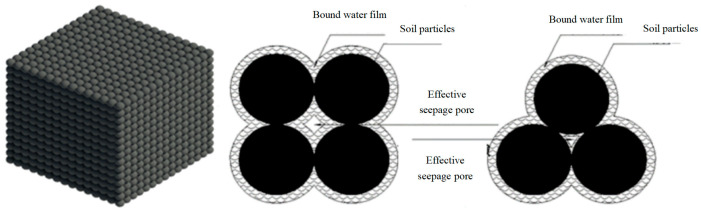
Soil particle distribution and capillary cross section model.

**Figure 2:**
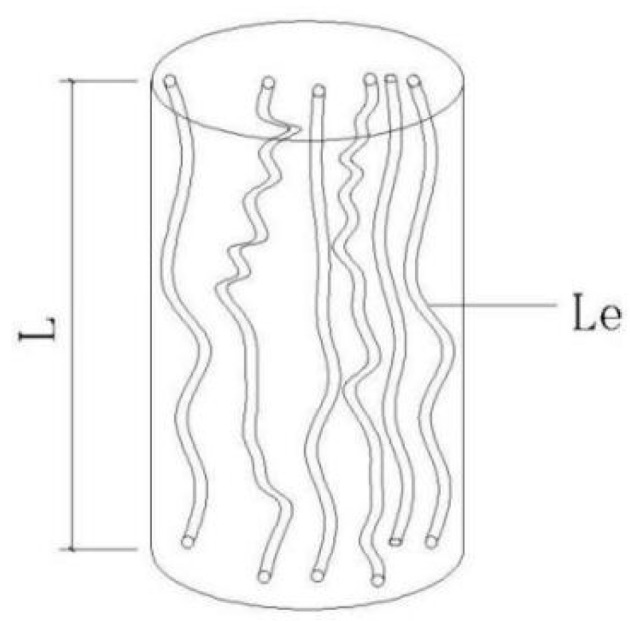
Schematic diagram of clay equivalent seepage capillary pipeline.

Therefore, to facilitate the derivation of the seepage theoretical model of saturated clay, this paper makes the following assumptions:

(1) It is assumed that the saturated clay is composed of ideal spheres with continuous distribution and uniform particles, as shown in Figure 1;

(2) It is assumed that the pores within the saturated clay are connected to form a seepage passage, which can be generalized as a flow system composed of a large number of capillary tubes of the same length and diameter with a certain degree of tortuosity, as shown in Figure 2;

(3) It is assumed that the equivalent seepage capillaries in saturated clay have the same hydraulic radius, specific surface area, and internal volume;

(4) It is assumed that the equivalent seepage capillaries in saturated clay are independent of each other and do not affect one another.

**(2)**  **Establishment of theoretical calculation formula for permeability coefficient of saturated clay**

According to practical engineering experience, the seepage velocity of water in saturated clay is not more than 10^−6^ cm/s, so the seepage form of water in saturated clay is laminar flow. The seepage model in this paper is based on the equivalent independent seepage capillary pipeline model of saturated clay. Based on Poiseuille’s law, the water flow rate Q of a single equivalent seepage capillary tube in saturated clay is determined as follows:

(1) $$Q=\frac{\gamma_{w}\pi r_{e}^{4}}{8\eta }\cdot \frac{\Delta p}{L_{e}}$$

where $r_{e}$ is the equivalent radius of a clay seepage capillary (m); η is the dynamic viscosity of water (kPa·s); $L_{e}$ is the equivalent bending length of a clay seepage capillary (m); Δp is the head difference between two ends of the capillary (m); and $\gamma_{w}$ is the unit weight of water (kN/m^3^).

In fact, the seepage network in clay is formed by complicated and interconnecting pore channels, and the perfectly straight pore channels account for a very small proportion of these in clay. Carman (1939) generalizes them as capillary tubes with a certain degree of tortuosity [21]. According to the analysis of a large number of indoor seepage test results, it is found that the actual seepage velocity of clay is less than the seepage velocity calculated by the capillary model of clay equivalent seepage. In this paper, according to the concept of pipeline curvature, the zigzag degree of the clay equivalent seepage capillary pipe is quantitatively parameterized by curvature. Therefore, the actual length of the equivalent seepage capillary in clay along the seepage direction is as follows:

(2) $$L_{e}=\tau L$$

where τ is the curvature of equivalent seepage capillary in clay; and *L* is the straight length of equivalent seepage capillary in clay along the direction of water flow (m).

Bruggeman (1935) assumes that clay particles are ideal spherical particles, and clusters are structured to form clay bodies [22]. Based on the research and analysis of a large number of microscopic experimental results of clay, a statistical formula for determining the curvature of the equivalent seepage capillary channel of clay based on the effective void ratio of clay is established:

(3) $$\tau =n_{e}^{-0.5}$$

where $n_{e}$ is the effective porosity of the clay.

The effective porosity of clay is determined according to the conversion of three indexes of soil:

(4) $$n_{e}=\frac{e_{e}}{1+e}$$

where $e_{e}$ is the effective void ratio of clay and *e* is the total void ratio of clay.

The equivalent bending length of capillary tube with equivalent seepage flow in clay is obtained by substituting Formulas (3) and (4) into Formula (2):

(5) $$L_{e}=L{\left(\frac{e_{e}}{1+e}\right)}^{-0.5}$$

Formula (5) is substituted into Formula (1) to obtain the water flow *Q* of the cross section of a single equivalent capillary tube in clay per unit time:

(6) $$Q=\frac{\gamma_{w}\pi r_{e}^{4}}{8\eta }\cdot \frac{\Delta p}{L{\left(\frac{e_{e}}{1+e}\right)}^{-0.5}}$$

The macro hydraulic gradient i of the clay is as follows:

(7) $$i=\frac{\Delta p}{L}$$

Formula (7) is substituted into Formula (6) to obtain the water flow rate *Q* of the cross section of a single clay equivalent seepage capillary per unit time:

(8) $$Q=\frac{\gamma_{w}\pi r_{e}^{4}}{8\eta }\cdot {\left(\frac{e_{e}}{1+e}\right)}^{0.5}\cdot i$$

According to the hypothesis of the model, the seepage passage in clay is composed of independent equivalent seepage capillaries, and the total seepage quantity of the clay cross section per unit time is equal to the total seepage quantity of the clay equivalent seepage capillaries cross section. Based on the cross section of clay, effective void ratio, and the cross section radius of clay equivalent seepage capillary pipe, the total number *N* of equivalent capillary tubes in the clay capillary model is established:

(9) $$N=\frac{n_{e}A}{\pi r_{e}^{2}}=\frac{e_{e}A}{(1+e)\pi r_{e}^{2}}$$

where *A* is the cross sectional area of the saturated clay sample.

The total water flow per unit time through the cross section of the saturated clay is determined according to the calculation of Formula (10):

(10) $$Q_{Z}=QN=\frac{\gamma_{w}\pi r_{e}^{4}}{8\eta }\cdot {\left(\frac{e_{e}}{1+e}\right)}^{0.5}\cdot i\cdot \frac{e_{e}A}{(1+e)\pi r_{e}^{2}}=\frac{\gamma_{w}r_{e}^{2}}{8\eta }{\left(\frac{e_{e}}{1+e}\right)}^{1.5}\cdot iA$$

The cross section of seepage passage in clay is different in shape and size. In this paper, based on the basic concept of hydraulic radius, the sizes of seepage channels with different shapes and scales in saturated clay are averaged. The hydraulic radius $R_{H}$ is expressed by the following formula:

(11) $$R_{H}=\frac{A_{f}}{S_{f}}$$

where $A_{f}$ is the flow area of flow trough in clay and $S_{f}$ is the wet perimeter of the flow trough in the clay.

Based on the theory of effective pore specific surface area and effective void ratio, the effective hydraulic radius of equivalent porous capillary in saturated clay can be obtained from Formulas (12) and (13):

(12) $$R_{H}^{e}=\frac{\pi r_{e}^{2}}{2\pi r_{e}}=\frac{r_{e}}{2}$$

(13) $$R_{H}^{e}=\frac{A_{f}^{e}}{S_{f}^{e}}=\frac{A_{f\cdot L_{e}}^{e}}{S_{f}^{e}\cdot L_{e}}=\frac{V_{s}\cdot e_{e}}{V_{s}\cdot S_{e}}=\frac{e_{e}}{S_{e}}$$

where $A_{f}^{e}$ is the effective flow area of equivalent seepage capillary in saturated clay and $S_{e}$ is the effective wet circumference of equivalent seepage capillary in saturated clay. That is, the effective pore specific surface area of soil particles in saturated clay.

Simultaneous Formulas (12) and (13) derive the equivalent radius of capillary tubes for saturated clay seepage:

(14) $$r_{e}=\frac{2e_{e}}{S_{e}}$$

Substituting Formula (14) into Formula (10), the total water flow per unit time of saturated clay through its cross section is as follows:

(15) $$Q_{Z}=\frac{\gamma_{w}e_{e}^{3.5}}{2\eta S_{e}^{2}(1+e{)}^{1.5}}\cdot iA$$

According to Darcy’s law, the total water flow per unit time of saturated clay is as follows:

(16) $$Q_{Z}=kiA$$

where k is the permeability coefficient of saturated clay.

The permeability coefficient of saturated clay can be obtained by simultaneous Formulas (15) and (16):

(17) $$k=\frac{\gamma_{w}e_{e}^{3.5}}{2\eta S_{e}^{2}(1+e{)}^{1.5}}$$

**Figure 3:**
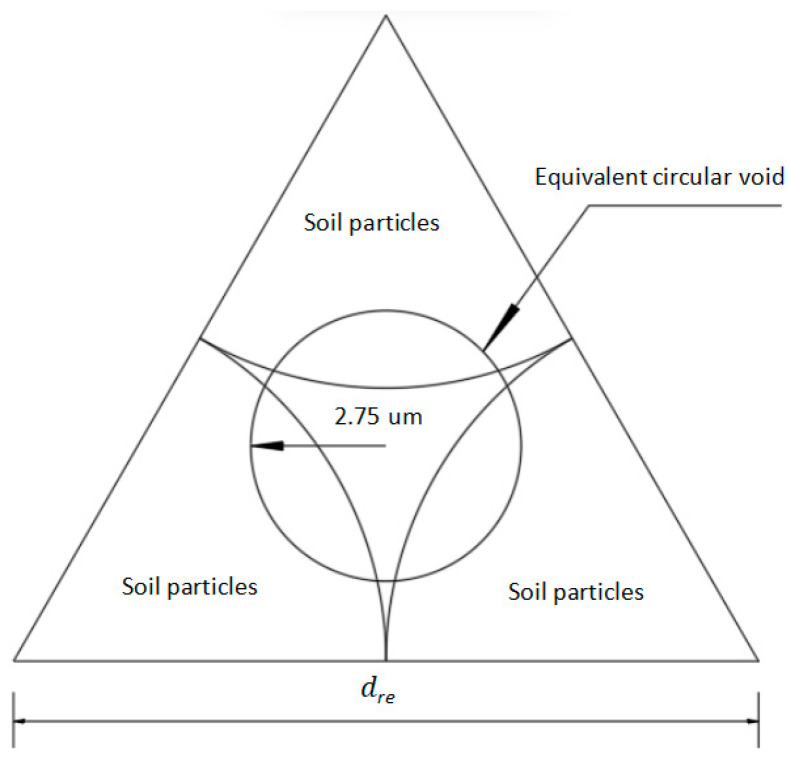
Equivalent pore radius of soil.

The seepage of saturated clay indicates the flow of water in the connected pore channel of saturated clay. Combined with the theory of hydrodynamics, in the process of seepage, water is mainly affected by the dynamic viscous resistance of water and the friction resistance of the channel wall, and the dynamic viscous resistance of water is determined by the dynamic viscosity coefficient of water. The seepage resistance of the clay pore channel to water is positively correlated with the inner surface area of the clay pore channel. The internal surface area of clay channels in clay equals the effective pore surface area of clay particles. Therefore, the effective pore specific surface area of clay is used to characterize the internal surface area of clay pore channels. Studies by Xie [23] demonstrate that the pore distribution in clay soils exhibits self-similarity, i.e., a fractal structure. Consequently, the fractal dimension from fractal theory can be employed to characterize the microstructure of soil. Based on geometric fractal theory, Equation (18) is used to determine the fractal dimension of clay particles, while Equation (19) is applied to calculate the effective pore specific surface area of clay particles in saturated soil. The theoretical formula is as follows:

(18) $$D=3-\frac{\mathrm{lg}m(d_{1})-\mathrm{lg}m(d_{2})}{\mathrm{lg}(d_{1})-\mathrm{lg}(d_{2})}$$

(19) $$S_{e}=\frac{6(\frac{1+e-e_{e}}{1+e})(3-D)(d_{\max}^{2-D}-d_{\min}^{2-D})}{(\frac{e_{e}}{1+e})(2-D)(d_{\max}^{3-D}-d_{\min}^{3-D})\left[\frac{e_{e}}{1+e}-0.34\frac{d_{re}^{3-D}-d_{\min}^{3-D}}{d_{\max}^{3-D}-d_{\min}^{3-D}}(\frac{1+e-e_{e}}{1+e})\right]}$$

where *D* is the fractal dimension of the particle size of clay; $m(d_{1})$ and $m(d_{2})$, respectively, represent the number of particles with diameter less than $d_{1}$ and $d_{2}$; $d_{1}$ and $d_{2}$, respectively, represent a group of soil particles in the clay particle gradation curve; and $d_{max}$, $d_{min}$, and $d_{\mathrm{re}}$ represent maximum and minimum soil particle size in clay and the effective seepage limit soil particle size. As demonstrated by He’s research [24], soil particles with pore radii below 2.75 um exhibit no seepage. The irregular pores are modeled as spherical (Figure 3), with an equivalent radius of 2.75 um. Using geometric relationships, the effective seepage limit for soil particles is calculated as $d_{\mathrm{re}}=2.42\times 10^{-5}$ m.

Ren et al. (2016) studied the ratio of the volume of flowing water to the volume of non-flowing water in saturated clay, concluding that the ratio is a positive power function with the void ratio of saturated clay [25]. But the main body of saturated clay seepage is free gravity water and partially weak combined water, so the effective void ratio in saturated clay is equal to the ratio of flowing water to soil particle. Then, based on the total void ratio of clay, the effective void ratio of clay is derived as follows:

(20) $$e_{e}=\frac{e}{2-(\frac{e}{1+e}{)}^{m}}$$

where *m* is a constant related to the properties of clay, and its value is positively correlated with the average particle size of clay. The average particle size of clay samples in this paper is 0.01 mm. According to the research results of Xiao [26], the value of m can be taken as 1.

### 2.2. Theoretical Calculation Formula of Permeability Coefficient of Saturated Clay Before Revision

When using saturated clay void ratio and total pore specific surface area parameters, the theoretical calculation formula of the equivalent capillary permeability coefficient of saturated clay is as follows:

(21) $$S_{e}=\frac{6(\frac{1}{1+e})(3-D)(d_{\max}^{2-D}-d_{\min}^{2-D})}{(\frac{e}{1+e})(2-D)(d_{\max}^{3-D}-d_{\min}^{3-D})\left[\frac{e}{1+e}-0.34\frac{d_{re}^{3-D}-d_{\min}^{3-D}}{d_{\max}^{3-D}-d_{\min}^{3-D}}(\frac{1}{1+e})\right]}$$

(22) $$k=\frac{\gamma_{w}e^{3}}{2\eta (1+e)S^{2}}$$

### 2.3. Modified Kozeny–Carman Permeability Equation

The modified Kozeny–Carman permeability equation is one of the most widely used theoretical calculation formulas of permeability coefficient in porous media.

(23) $$k=\frac{c\rho_{w}e^{3}}{\eta (1+e)S^{2}}$$

where *c* is the coefficient related to the shape of clay particles and the actual seepage direction, the general value is 0.125; $\rho_{w}$ is the density of water ($g/cm^{3}$); and η is the dynamic viscosity coefficient of water ($g\cdot s/cm^{2}$).

## 3. Clay Permeability Test

### 3.1. Grain Size Analysis of Test Soil Sample

**Figure 4:**
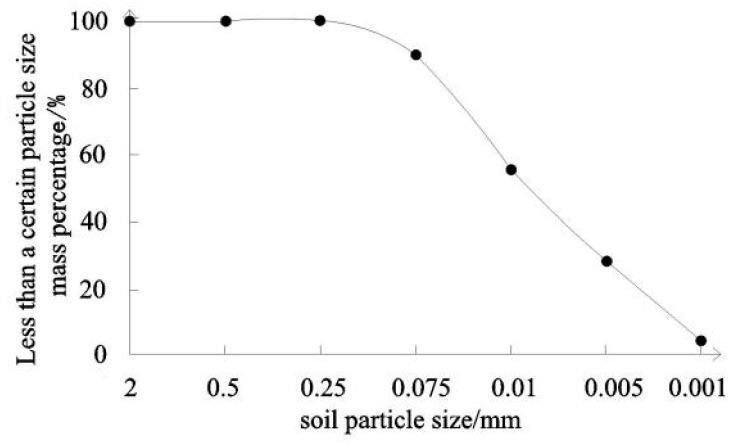
Gradation curve of clay samples.

The remolded clay is selected as the sample soil, and according to the Chinese standard of geotechnical test method (GB/T50123-2019 [27]), the density meter test is used to analyze the particles, and the particle grading curve is drawn according to the test results, as shown in Figure 4.

### 3.2. Preparation of Clay Specimens

**Figure 5:**
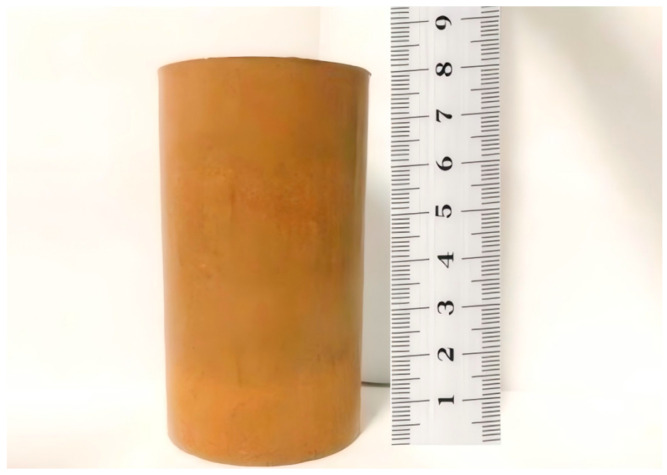
Compacted clay specimen.

In this experiment, a cylindrical remolded clay sample with a cross section diameter of 3.91 cm and a height of 8 cm was selected. The experiment was carried out in the environment of 20 degrees. According to the manufacturing steps of compacted soil samples in standard for geotechnical test methods (GB/T50123-2019) by using compaction instrument, five soil samples with different void ratios (Figure 5) were made. The values of the void ratios are shown in Table 1.

**Figure 6:**
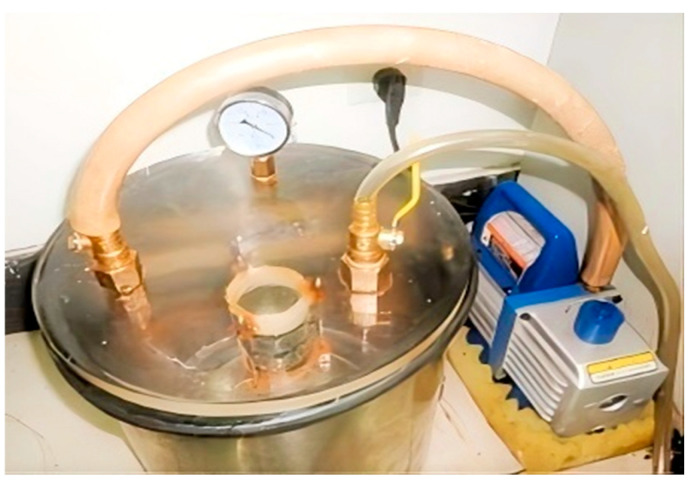
Vacuum saturation treatment of soil samples.

After compaction, place a piece of filter paper and a permeable stone at each end of the test soil sample, and record the sticker accurately. According to the permeability and structure of the test soil, the vacuum saturation method is chosen to treat the test soil saturation, as shown in Figure 6.

The saturated clay sample is loaded into the permeameter, and the saturated clay sample continues to be saturated for 12 h after venting. The permeability test of the variable head is carried out by setting the initial head difference of 2 m. After the seepage is stable, the water temperature is measured and the reading recorded. The specific test steps refer to Article 16.3 of the Chinese Standard for Geotechnical Test methods (GB/T50123-2019).

### 3.3. Results of Penetration Test

According to the recorded data of the variable water head permeability test of each clay sample, the permeability coefficients of five clay samples under different void ratios were calculated and analyzed.

According to Table 2, the order of magnitude of the permeability coefficient of clay is basically stable in the range of 10^−6^~10^−7^ cm/s, and there is a positive nonlinear relationship between permeability coefficient and void ratio.

## 4. Verification and Analysis of Theoretical Formula for Permeability Coefficient of Saturated Clay

According to the above clay particle size test results, it is known that the maximum size of soil particles in clay is 0.05 cm, but the minimum particle size of the clay cannot be determined from the results of the clay particle test. According to the research results in *Introduction to Colloid Chemistry* [28], when the size of a soil particle is less than 1 × 10^−5^ cm, the existence form of the soil particle is a colloid particle, so the minimum size of a soil particle in clay is 1 × 10^−5^ cm. The dynamic viscosity coefficient of the water is 1.01 × 10^−6^ kPa·s, as the test temperature of the variable head penetration test is 20 °C. *m* is a constant related to the nature of the clay. The average particle size of clay sample in this paper is 0.01 mm, and it is suggested that *m* = 1. The fractal dimension *D* of clay is calculated in Table 3.

By using the above parameters and the theoretical calculation formula of the equivalent capillary permeability coefficient of saturated clay and the modified Kozeny–Carman equation, the theoretical permeability coefficients of each clay sample are determined. We carried out comparison and analysis of the results of permeability tests with variable head, and the results are shown in Table 4.

**Figure 7:**
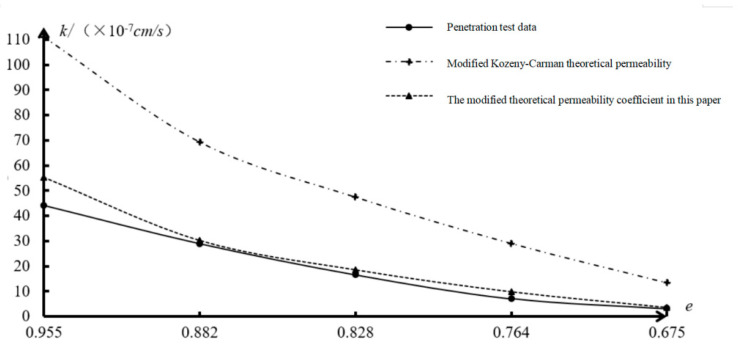
Curve of saturated clay test and theoretical permeability coefficient.

**Figure 8:**
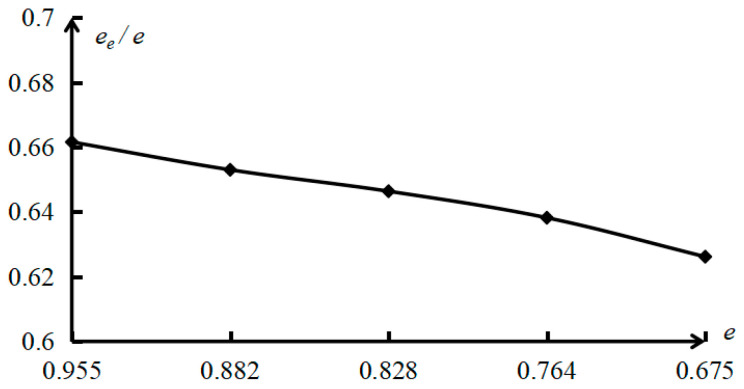
The ratio curve of the effective void ratio to the total void ratio of saturated clay.

**Figure 9:**
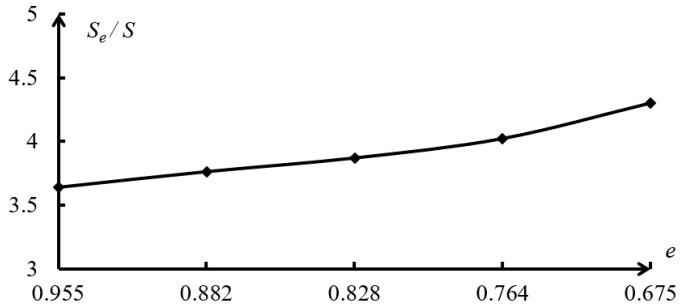
The ratio curve of effective pore specific surface area to total pore specific surface area of saturated clay.

Based on the study and analysis of the above experimental permeability coefficient and theoretical permeability coefficient, it is found that the clay permeability coefficient determined according to the theoretical formula of saturated clay permeability coefficient before correction in this paper is 95.733–171.729 times the test permeability coefficient, and the error is too large. The reference value is extremely low. The theoretical calculation formula of the permeability coefficient of clay equivalent capillary pipe and the modified Kozeny–Carman equation has a small error with the experimental permeability coefficient, so it is of great value in engineering application and theoretical research. According to the analysis results of the clay permeability coefficient of the above three kinds of soils, the curve of saturated clay test and theoretical permeability coefficient is drawn in Figure 7. Based on the modified equivalent capillary permeability model and the physical and mechanical parameters of saturated clay samples, the variation in effective void ratio and effective void specific surface area of saturated clay is analyzed, the ratio curves of effective void ratio to total void ratio of saturated clay and the ratio curves of effective void specific surface area to total void specific surface area of saturated clay are shown in Figure 8 and Figure 9, respectively.

According to the test permeability coefficient and theoretical permeability coefficient results of saturated clay samples in Table 4 and Figure 7, the test permeability coefficient is taken as the benchmark term, the two theoretical permeability coefficients are compared for relative error analysis, and the relative error of the modified Kozeny–Carman equation is 1.39–3.29. The calculation relative error of the modified model established in this paper is 0.04–0.39, which shows that the theoretical formula for the permeability coefficient of saturated clay and the modified Kozeny–Carman equation established in this paper are more reasonable to calculate the permeability coefficient of saturated clay. Moreover, the error of the modified model established in this paper is smaller, and the index data of clay particle diameter and mass percentage, void ratio, water weight, and dynamic viscosity coefficient are easily obtained through laboratory tests. The model has certain reference value for the seepage analysis and anti-seepage design of civil, geotechnical, geological and hydraulic engineering and the theoretical study of seepage consolidation settlement of saturated clay.

From the comparative analysis in Table 4 and Figure 7, it can be seen that the modified Kozeny–Carman equation uses the saturated clay void ratio and pore specific surface area. However, a Kozeny–Carman constant, which is related to the grain shape and the actual seepage direction of saturated clay, has not yet formed an accurate theoretical formula and is only a comprehensive empirical value with no practical physical significance. Therefore, the calculation accuracy of the modified Kozeny–Carman equation is lower than that of the modified saturated clay equivalent capillary permeability coefficient model established in this paper. With the decrease in the void ratio, the error of the theoretical permeability coefficient is gradually reduced, which indicates that the modified model is more suitable for the saturated clay with small void ratio. According to the study of the seepage mechanism, with the decrease in void ratio of saturated clay, the compactness of soil mass increases, and the void ratio of seepage in saturated clay increases. The theoretical formula of the permeability coefficient of saturated clay in this paper is modified on the basis of decreasing void ratio of saturated clay particles and increasing effective specific surface area and capillary diameter length; therefore, the calculated results are more suitable for the saturated clay with small porosity.

As can be seen from Table 4 and Figure 8 and Figure 9, the ratio of the effective void ratio to the total void ratio of saturated clay is positively related to the total void ratio of saturated clay, indicating that with the decrease in the total void ratio of saturated clay, the void fraction in saturated clay increases not only in quantity but also in percentage of total void fraction. The effective void fraction of saturated clay is about 0.6~0.7 times the total void fraction. In addition, although the effective pore specific surface area and total pore specific surface area of saturated clay are both negatively correlated with the total void ratio of saturated clay, their increases are different. As the void ratio of saturated clay decreases, not only does the effective pore specific surface area of saturated clay increase, but also the ratio of the effective pore specific surface area to the total pore specific surface area of saturated clay rises rapidly. It shows that the increase in the ineffective pore specific surface area in this saturated clay sample decreases and is smaller than the increase in the effective pore specific surface area of the saturated clay. The analysis of the reasons shows that with the decrease in the total void ratio of saturated clay, it is difficult to compress ineffective unconnected pores, and the connected pores are mainly compressed pores, resulting in a decrease in the cross sectional diameter of the saturated clay equivalent seepage capillary and an increase in the specific surface area of the pores. Combined with Formula (14), it is further demonstrated that the cross sectional diameter of the saturated clay equivalent seepage capillary has a positive correlation with the saturated clay void ratio, and a negative correlation with the saturated clay effective pore specific surface area. Therefore, the effective pore specific surface area of saturated clay is larger than the total pore specific surface area, which is about 3–5 times the total pore specific surface area of saturated clay.

To sum up, the decrease in the total void ratio of saturated clay results in a decelerated decrease in the effective void ratio of saturated clay and the cross sectional diameter of the equivalent seepage capillary. This, in turn, leads to an accelerated increase in the effective pore specific surface area of saturated clay, which ultimately causes a nonlinear decrease in the permeability coefficient of saturated clay.

## 5. Conclusions

From the above analysis and research, the following conclusions can be drawn:

(1) In this paper, based on the equivalent seepage capillary curvature model and the relationship between the volume ratio of flowing water and non-flowing water, and combined with the effective void ratio theory of saturated clay, the actual seepage path tortuosity of saturated clay is quantified as a parameter related to the void ratio. A theoretical formula for determining the effective void ratio based on the total void ratio of saturated clay is established, which improves the accuracy of the seepage path length of saturated clay and the calculation efficiency of the effective void ratio of saturated clay.
(2) Based on the theory of effective void ratio and effective void specific surface area of saturated clay, and combined with the basic physical and mechanical parameters of saturated clay determined by laboratory geotechnical tests, a theoretical formula for the permeability coefficient of saturated clay is established. The seepage test results of saturated remodeled clay samples verify the rationality and accuracy of the above theoretical formula for the saturated clay permeability coefficient. The error accuracy between the theoretical permeability coefficient value and the measured permeability coefficient value of saturated clay meets the engineering requirements. This not only reduces the work difficulty but also improves the calculation accuracy, and has broad practical engineering application prospects.
(3) By analyzing the variation law of the effective void ratio and effective pore specific surface area of saturated clay with the total void ratio of saturated clay, the nonlinear law of the permeability coefficient of saturated clay with the void ratio is explained. Combined with the indoor permeability test results of saturated clay, it is proved that the effective pore specific surface area ratio in saturated clay seepage analysis is superior to the traditional specific surface area (the ratio of soil particle surface area to soil particle volume).

## 6. Limitations and Future Prospects

(1) The model is currently validated on a single clay type using five samples varying only in void ratio. Future work will expand validation to additional clay types, enhancing the model’s applicability across diverse geological conditions.
(2) Due to the limitations of the experimental conditions of this team, conventional permeameter equipment was used, and factors such as the size of pores, connectivity, and micro-forces between particles in saturated clay were not studied. Therefore, the model established in this paper has certain limitations and a certain degree of error, and is mainly applicable to saturated yellow clay. In the next step, our team will conduct further exploration.

## Figures and Tables

**Table 1:** Void ratio of cohesive soil samples.

Sample Number	Y-1	Y-2	Y-3	Y-4	Y-5
Void ratio (*e*)	0.955	0.882	0.828	0.764	0.675

**Table 2:** Permeability coefficient of clayey soil samples under different void ratios.

Sample Number	Y-1	Y-2	Y-3	Y-4	Y-5
Void ratio (*e*)	0.955	0.882	0.828	0.764	0.675
Permeability coefficient/(×10^−7^ m/s)	44.132	28.958	16.578	7.039	3.131

**Table 3:** Fractal dimension of clay sample *D*.

Parameter Index	*d*_1_/cm	*d*_2_/cm	*m* (*d*_1_)/%	*m* (*d*_2_)/%	*D*
Clay soil	0.0075	0.001	90.2	55.6	2.76

**Table 4:** Comparison and analysis of permeability coefficient calculation results with experimental results.

Sample Number	Y-1	Y-2	Y-3	Y-4	Y-5
Void ratio *e*	0.955	0.882	0.828	0.764	0.675
Effective void ratio *e*_e_	0.632	0.576	0.535	0.488	0.423
Specific surface area of effective void ratio *S*_e_/(cm^2^/g)	256,498.669	303,519.033	348,315.358	417,379.264	559,289.406
Test permeability/(×10^−7^ cm/s)	44.132	28.958	16.578	7.039	3.131
Theoretical permeability coefficient before correction/(×10^−7^ cm/s)	4438.558	2772.230	1897.149	1162.178	537.685
Modified Kozeny–Carman Theoretical permeability coefficient/(×10^−7^ cm/s)	110.964	69.306	47.429	29.054	13.442
The revised theoretical permeability coefficient is presented in this paper/(×10^−7^ cm/s)	55.187	30.181	18.518	9.818	3.584

## Data Availability

The original contributions presented in this study are included in the article. Further inquiries can be directed to the corresponding authors.
